# Influence factor analysis and prediction model of successful application of high-volume Foley Catheter for labor induction

**DOI:** 10.1186/s12884-023-06101-7

**Published:** 2023-11-09

**Authors:** Jia Wang, Yu Cao, Lu Chen, Yan Tao, Huanhuan Huang, Chunju Miao

**Affiliations:** 1 Department of Gynecology and Obstetrics, Changshu No.1 People’s Hospital, Suzhou, 215500 China; 2 School of Biotechnology and Food Engineering, Changshu Institute of Technology, Suzhou, 215500 China

**Keywords:** Labor induction, Foley catheter, Water balloon, Prediction model, Nomogram

## Abstract

**Background:**

This study aimed to establish a clinical-based nomogram for predicting the success rate of high-volume Foley catheterization for labor induction.

**Methods:**

This retrospective study included 1149 full-term pregnant women who received high-volume Foley catheterization for labor induction from January 2019 to December 2021 in Changshu No.1 People’s Hospital. Univariate and multivariate logistic regression analyses were performed, in which the labor induction success was set as dependent variables and the characteristics (including age, height, weight, BMI, gravidity, parity, gestational age, uterine height, abdominal circumference, cervical Bishop score, amniotic fluid index, cephalic presentation, neonatal weight, pregnancy complications, etc.) were set as independent variables. A nomogram scoring model was established based on these risk factors, and a calibration curve was plotted to verify the predictive accuracy of the model.

**Results:**

The success rate of labor induction was 83.55% (960/1149). Univariate analysis revealed that the risk factors associated with the success rate of high-volume Foley catheterization for labor induction were height, pregnancy, birth, age, weight, BMI, uterine height, abdominal circumference, and hypertension. Multivariate logistic regression analysis showed that age (OR = 0.950; 95% CI: 0.904 ~ 0.998), height (OR = 1.062; 95% CI: 1.026 ~ 1.100), BMI (OR = 0.871; 95% CI: 0.831 ~ 0.913), and parity (OR = 8.007; 95% CI: 4.483 ~ 14.303) were independent risk factors for labor induction success by high-volume Foley catheterization. The area under the curve (AUC) of the receiver operating characteristic (ROC) curve in the prediction model was 0.752 (95% CI 0.716 ~ 0.788). A nomogram was constructed based on the final multivariate analysis with a corrected C-index of 0.748, which indicated that the model was calibrated reasonably.

**Conclusion:**

Four risk factors were used to construct a nomogram to evaluate the success rate of high-volume Foley catheterization for labor induction. The nomogram provides a visual clinical tool to assist in the selection of the most appropriate mode of labor induction for pregnant women of different risk levels.

## Introduction

Labor induction is a common obstetric intervention that refers to the process of expediting delivery. A rapid increase in the rate of labor induction has been observed over the past few years [1, 2]. Cervical maturation is one of the most important events during labor induction, and developing safe and efficient strategies to promote cervical maturity is crucial. Compared to conventional strategies such as the administration of prostaglandins, mechanical labor induction has a lower incidence of uterine overcontraction, thereby decreasing complications such as placental abruption and fetal intrauterine distress [3, 4]. The Foley catheter is frequently used in mechanical labor induction. This method offers various advantages, including safety, effectiveness, patient satisfaction, and low cost [5]. As a result, the Foley catheter has become a globally representative mechanical induction method for cervical maturation.

In this study, a large-volume Foley catheter (60 mL) was used for labor induction in the third trimester of pregnancy. The potential risk factors associated with the use of the Foley catheter were analyzed based on the clinical characteristics of pregnant women undergoing labor induction. Furthermore, a logistic regression equation was used to determine the influence of the Foley catheter on labor induction. In addition, nomograms were generated to construct predictive models, which could provide clinical guidance for labor induction in women of various risk levels in the third trimester of pregnancy.

## Materials and methods

### Participants

This was a retrospective study that included a total of 1177 women who were hospitalized in the second-class hospital Changshu First People’s Hospital between January 1, 2019, and December 31, 2021. All participants were full-term and met the inclusion and exclusion criteria for receiving a large-volume (60 mL) Foley catheter for labor induction. The operators who performed the procedure had extensive experience in placement.

**Fig. 1:**
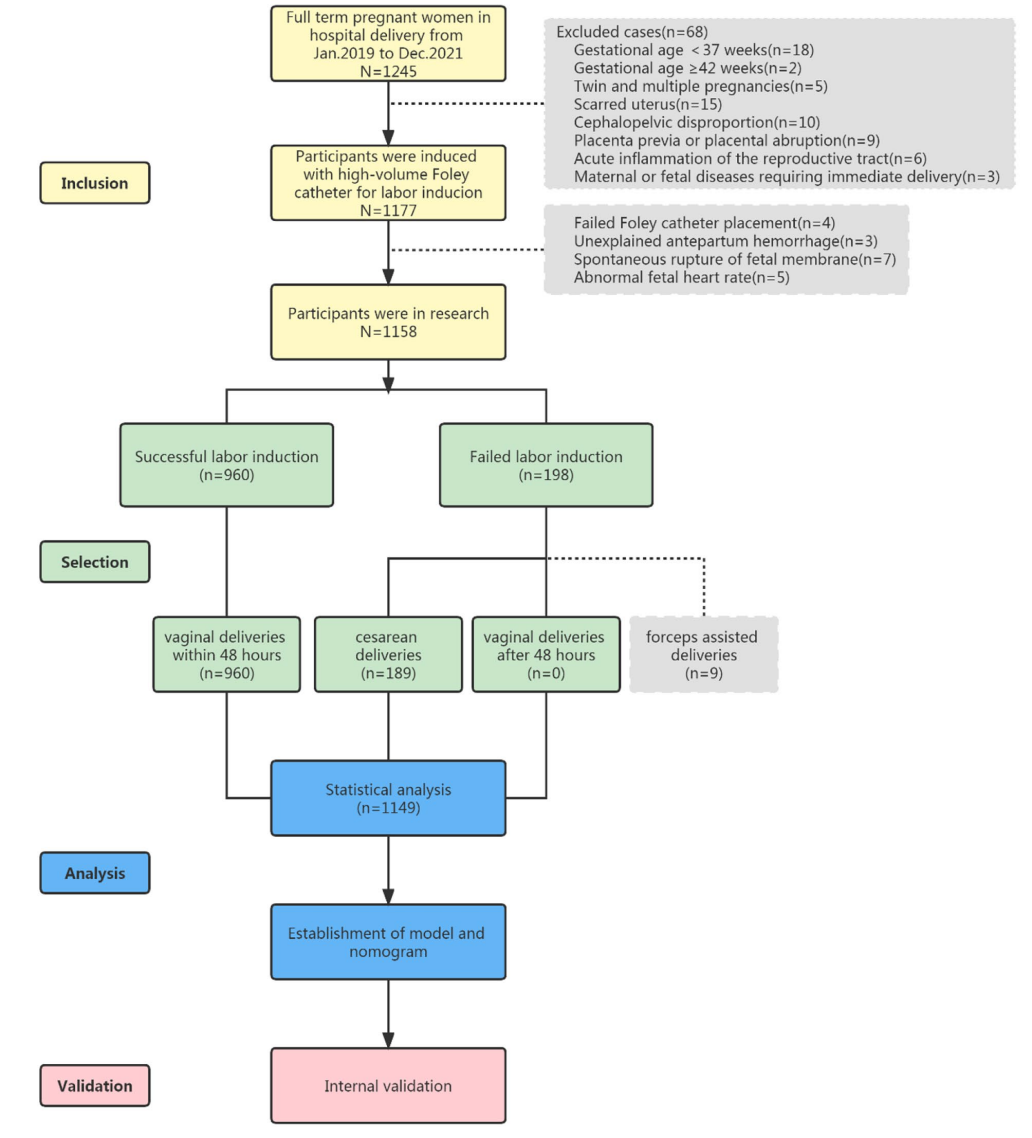
Study flowchart

Among the 1,177 women who received a large-volume (60 mL) Foley catheter for labor induction, four cases had a failed placement, three cases had unexplained antepartum hemorrhage after placement, five cases had abnormal fetal heart rate after placement, and seven cases had spontaneous rupture of fetal membrane after placement. Labor induction was successful in 960 cases of vaginal delivery within 48 h after the placement of the high-volume Foley catheter. The cases in which labor was not induced by 48 h of Foley catheter placement were delivered by cesarean section. In this study, 189 patients underwent cesarean section and nine cases received forceps-assisted delivery. Finally, a total of 1,149 cases were included in the analysis, yielding a success rate of labor induction of 83.55% (960/1,149). The flow chart is displayed in Fig. 1.

### Study outcomes

The primary outcome of the study was the success rate of labor induction. Successful labor induction was defined as vaginal delivery within 48 h after the placement of the high-volume Foley catheter. Vaginal deliveries that occurred after 48 h, as well as forceps-assisted deliveries and cesarean deliveries, were defined as failed induction of labor.

### Inclusion and exclusion criteria

The inclusion criteria for the study were as follows: (1) pregnant women with singleton gestation; (2) gestational age ≥ 37 weeks; (3) cephalic presentation; (4) cervical Bishop score ≤ 6; (5) intact fetal membranes; and (6) complete medical records. The exclusion criteria were as follows: (1) gestational age < 37 weeks; (2) gestational age ≥ 42 weeks; (3) twin and multiple pregnancies; (4) scarred uterus; (5) cephalopelvic disproportion; (6) placenta previa or placental abruption; (7) acute inflammation of the reproductive tract; and (8) maternal or fetal diseases requiring immediate delivery.

### Operation flow and termination signal

The following steps were taken during the Foley catheter placement procedure: (1) Pregnant women were placed in the cystotomy position, and the vulva was routinely disinfected with 0.5% iodophor. Aseptic towels were placed and the cervix was exposed using a speculum. The vagina and cervix were then disinfected with 0.5% iodophor. (2) The Foley catheter was inserted into the cervix with oval forceps to a depth of approximately 6 cm, and 60 mL of physiological saline was injected into the end of the catheter. The catheter was gently pulled to bring the water balloon close to the orificium internum uteri. The end of the catheter was then attached to the inner thigh, and the patient was monitored for abdominal pain, vaginal bleeding, and fetal heart rate. (3) After 12 h of Foley catheter placement, the vulva was disinfected with 0.5% iodophor again and the cervical water balloon was removed if it had not fallen out by that time. Thereafter, cervical maturity was checked. (4) If the cervical Bishop score was ≥ 6 points, an artificial rupture of fetal membranes was performed, and the woman was observed for 1 h. If the woman still had no contractions, she was given a drip of 500 mL 5% glucose solution mixed with oxytocin 2.5 u, starting at 6 drops per minute. The infusion rate was gradually increased until effective uterine contractions were achieved. If the cervical Bishop score was < 6 points, the artificial rupture of fetal membranes was not performed, and the woman was given a drip of 500 mL 5% glucose solution mixed with oxytocin 2.5 u, starting from 6 drops per minute. The infusion rate was gradually increased until effective uterine contractions were achieved. The session was terminated in any of the following cases: (1) unexplained abnormal prenatal bleeding after placement; (2) spontaneous rupture of fetal membranes; or (3) detection of an abnormal fetal heart rate.

### Ethics approval

The Ethics Committee of Changshu No.1 People’s Hospital approved the study’s procedures (reference number 2019 Ethics Review (Declaration) Batch No. 13). All participants provided written informed consent, and the ethics committee approved the consent procedures.

### Statistical analysis

The statistical analysis was performed using SPSS 17.0 software. The chi-square test of proportions was used to analyze categorical variables affecting the success of labor induction. Mean and standard deviation with 95% CIs were used for the t-test. Univariate logistic regression analysis was performed and significant indicators were further analyzed by multinomial logistic regression (Enter Method). Accordingly, an ROC curve was drafted to analyze the predictive value of the model. Multivariate analysis was performed using R language statistical software. Thereafter, a nomogram was drafted based on the prediction model, and the prediction accuracy of the model was evaluated by the C index (equivalent to the area under the ROC curve). All internal validation work was conducted in the resampling data of Bootstrap. The calibration curve was drafted to obtain the calibrated C index.

## Results

### Characteristics of the participants and pregnancy outcomes

According to the medical records of the 1149 included women, the success rate of labor induction was 83.55% (960 / 1149). Table 1 shows the antenatal characteristics, indications for labor induction, and neonatal outcomes of both groups. Significantly lower age, weight, BMI, uterine height, and abdominal circumference were observed in the successfully induced labor group compared to the failed induction group (p < 0.05). In addition, the mean height (162 cm) was significantly higher in the successfully induced labor group than in the failed induction group (160 cm) (p = 0.002). Pregnant women with high cervical Bishop scores or a higher number of pregnancies and parities showed a significant advantage in labor induction (p < 0.05). We counted the indications for surgery, including cephalopelvic disproportion in 31.75%, fetal factors in 21.16%, no indication for cesarean section in 14.81%, suspected intrauterine infection in 9.52%, and abnormal labor in 8.99%.

**Table 1:** Demographic and clinical characteristics of the participants

	Labor induction Success (n = 960)	Failure (n = 189)	*p-value*
Characteristic			
Maternal age (years, mean ± SD)	26.69 ± 3.78	27.49 ± 4.04	0.007
Height (meter, mean ± SD)	1.62 ± 0.05	1.60 ± 0.48	0.002
Weight (kg, mean ± SD)	70.54 ± 9.26	73.52 ± 10.98	0.001
BMI (kg/m^2^, mean ± SD)	26.97 ± 3.15	28.53 ± 3.78	< 0.001
Gravidity (Median;IQR)	2;2	1;1	< 0.001
Parity (Median;IQR)	0;1	0;0	< 0.001
Gestational age (day, mean ± SD)	274.71 ± 13.23	275.96 ± 7.21	0.219
Uterine height (cm, mean ± SD)	35.00 ± 2.33	35.67 ± 2.23	0.002
Abdominal circumference (cm, mean ± SD)	100.39 ± 6.99	102.70 ± 7.70	< 0.001
Cervical Bishop score (mean ± SD)	4.61 ± 0.59	4.53 ± 0.56	0.049
Indications of induced abortion			
Decreased amniotic fluid (n) [%]	252[26.25]	41[21.69]	0.202
Oligohydramnios (n) [%]	56[5.83]	12[6.35]	0.738
GDM (n) [%]	147 [15.31]	37 [19.58]	0.158
ICP (n) [%]	34 [3.54]	6 [3.17]	1.000
HDP (n) [%]	48 [5.00]	21 [11.11]	0.002
Late term (41weeks or greater) (n) [%]	354[36.88]	64[33.86]	0.457
Fetal causes^a^ (n) [%]	40[4.17]	5[2.65]	0.414
Others^b^ (n) [%]	29[3.02]	3[1.59]	0.341
Neonatal outcome			
Neonatal weight (g, mean ± SD)	3348.65 ± 385.52	3405.03 ± 409.09	0.180
Apgar score >7 at 5 min(n) [%]	958[99.79]	189[100]	
Apgar score ≤ 7 at 5 min(n) [%]	2[0.21]	0	

^a^Fetal causes include: Fetal intrauterine growth restriction, suspected abnormalities in fetal heartbeat monitoring, umbilical cord factors

^b^Others include: Abnormal liver function, systemic lupus erythematosus, thrombophilia, renal disease

### Univariate and multivariate regression analysis

Various risk factors, including age, height, weight, BMI during labor, gravidity, parity, gestational age, uterine height, abdominal circumference, cervical Bishop score, vertex presentation, birth weight, and five indications for induction of labor (including decreased amniotic fluid and oligohydramnios, hypertensive disorders of pregnancy, intrahepatic cholestasis of pregnancy, gestational diabetes mellitus) were subjected to univariate regression analysis. Among the statistically significant factors, height, gravidity, and parity were positively correlated with the success rate of induction; in contrast, age, weight, BMI during labor, uterine height, abdominal circumference, and hypertensive disorders of pregnancy (HDP) were negatively associated with the success rate of labor induction. The OR values of parity and HDP were the largest and smallest, respectively (Table 2).

Furthermore, the variables were entered into the logistic multivariate regression analysis equation, including age, BMI, parity, and height. The regression equation was obtained as follows: logit (P) = 3.334 − 0.052 × Age − 0.138 × BMI + 2.080 × Parity + 0.061 × Height (cm). Among them, age and BMI were negatively associated with the success of induction, while parity and height were positively correlated (Table 2).

**Table 2:** Univariate and multivariate regression analysis of risk factors on the effect of labor induction utilizing high-volume Foley catheter

Characteristic	B	SE	Wald value	*p-value*	OR	95% CI
Univariate regression analysis						
Maternal age	-0.053	0.021	6.231	0.013	0.954	0.910 ~ 0.989
Height	0.053	0.017	9.892	0.002	1.055	1.020 ~ 1.090
Weight	-0.031	0.008	14.909	< 0.001	0.970	0.955 ~ 0.985
BMI	-0.135	0.023	33.374	< 0.001	0.874	0.834 ~ 0.915
Gravidity	0.505	0.097	27.260	< 0.001	1.657	1.371 ~ 2.003
Parity	1.850	0.273	45.931	< 0.001	6.357	3.723 ~ 10.851
Gestational age	-0.014	0.011	1.558	0.212	0.987	0.966 ~ 1.008
Uterine height	-0.113	0.034	10.820	0.001	0.893	0.835 ~ 0.955
Abdominal circumference	-0.049	0.012	17.243	< 0.001	0.952	0.931 ~ 0.957
Cervical Bishop score	0.228	0.133	2.923	0.087	1.256	0.967 ~ 1.631
Decreased amniotic fluid	0.251	0.191	1.720	0.190	1.285	0.883 ~ 1.869
Oligohydramnios	-0.090	0.329	0.075	0.784	0.914	0.480 ~ 1.740
Vertex presentation	0.175	0.216	0.658	0.417	1.919	0.780 ~ 1.819
Birth weight	-0.373	0.205	3.297	0.069	0.689	0.461 ~ 1.030
GDM	-0.297	0.204	2.123	0.145	0.743	0.498 ~ 1.108
ICP	0.113	0.450	0.063	0.801	1.120	0.463 ~ 2.706
HDP	-0.865	0.275	9.910	0.002	0.421	0.246 ~ 0.721
Multivariate regression analysis						
Age	-0.052	0.025	4.160	0.041	0.950	0.904 ~ 0.998
BMI	-0.138	0.024	32.711	< 0.001	0.871	0.831 ~ 0.913
Parity	2.080	0.296	49.409	< 0.001	8.007	4.483 ~ 14.303
Height	0.061	0.018	11.605	0.001	1.062	1.026 ~ 1.100

Abbreviations: SE, standard errors; B, beta coefficient; OR, odds ratio; CI, confidence interval

**Fig. 2:**
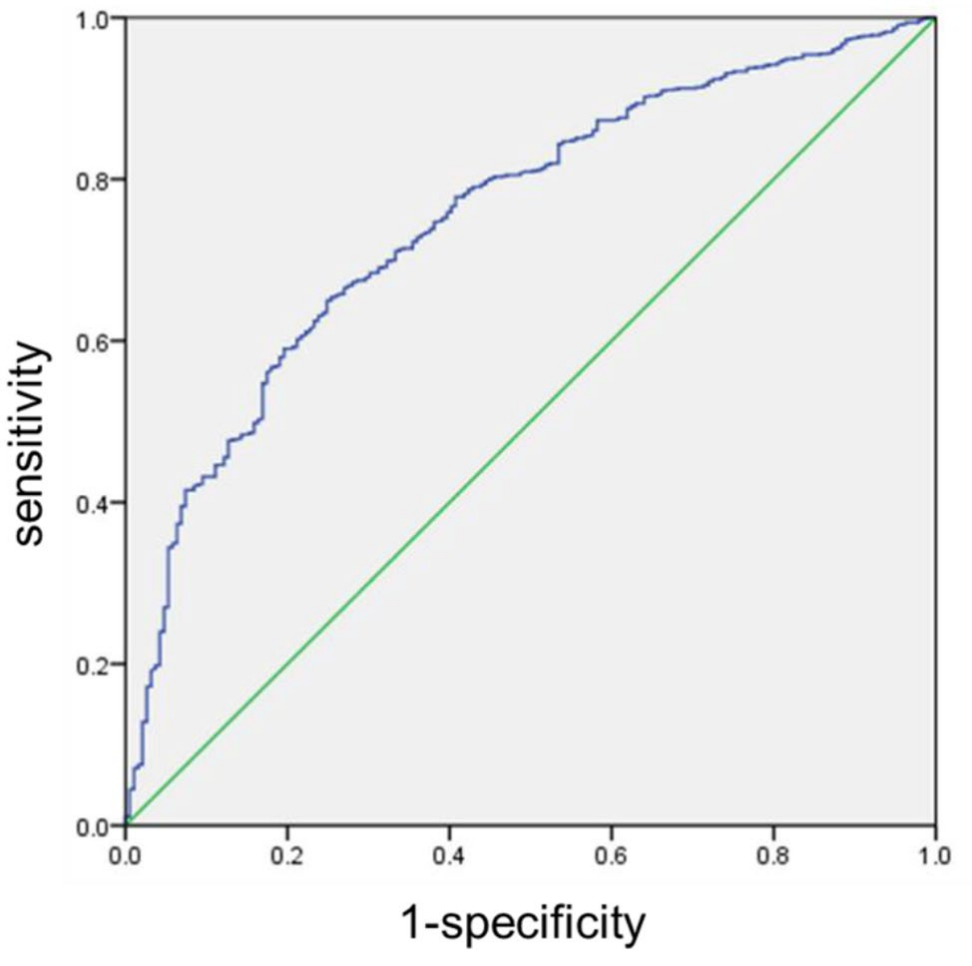
The ROC curve of the Logistic regression model

The area under the curve was calculated to be 0.752 (95% CI = 0.716–0.788). The optimal diagnostic cutoff value was determined to be 0.840 by the Youden index. Based on this threshold, 557 out of 593 cases in the regression model were correctly judged, yielding a positive prediction rate of 93.93%. Among the 556 cases that were predicted to fail in labor induction, 153 cases were correctly judged, with a negative prediction rate of 27.52%. The sensitivity was 0.580, the specificity was 0.810, and the accuracy was 0.618 (Fig. 2).

### A nomogram predicting the effect of Foley catheter on labor induction

**Fig. 3:**
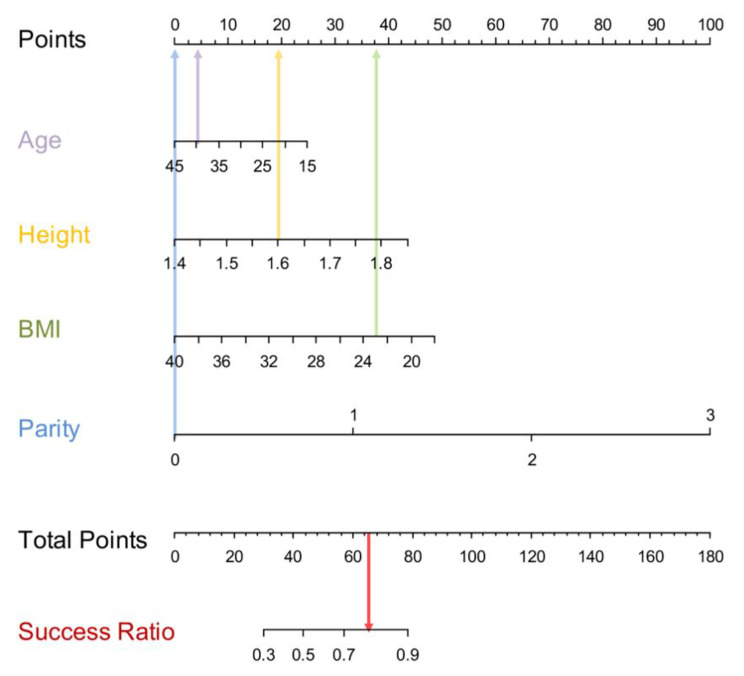
The nomogram including age, height, BMI, and birth time based on multivariate analysis

According to the results of the multivariate analysis, a nomogram was developed for clinical practice, which included age, height, BMI, and birth time (Fig. 3). The point axis at the top of the figure was used to assign scores for each variable, and the sum score was obtained. The total score corresponds to the predicted probability of successful labor induction.

**Fig. 4:**
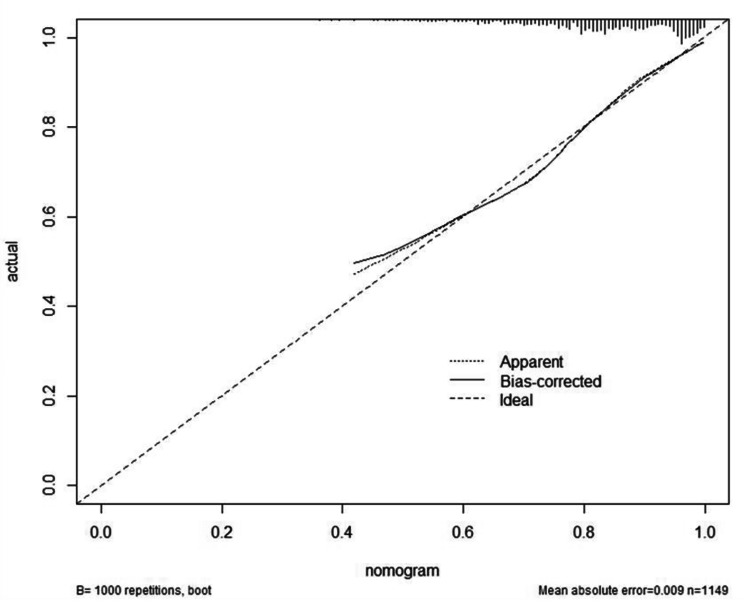
Modulation diagram of the calibration curve

The nomogram is used by adding the scores of every variable. The total points projected on the bottom scales indicate the probabilities of successful induction of labor. For example, a 30-year-old primipara with 160 cm height, 60 kg weight, and a BMI of 23.43 would have an integrated score of 66 (total points). Therefore, the probability of successful induction of labor is calculated to be approximately 80%. The overall prediction ability of the model is displayed in the nomogram. According to the original data, the nomogram-predicted model’s C-index was 0.752 (95% CI = 0.716–0.788). After validation by 1000 internal models, the calibrated C-index was 0.748. The limited reduction in C-index (0.004) indicated that the model was reasonably calibrated (Fig. 4).

The Bootstrap self-sampling method was used to verify the prediction effect of the model. The X-axis indicates the probability of the nomogram prediction, and the Y-axis indicates the actual operation probability. The Apparent dashed line represents the entire cohort, the Ideal dashed line represents the corresponding perfect prediction, and the Bias-corrected solid line represents the bias-corrected prediction by Bootstrapping (1000 replicates), which indicates the observed module performance.

## Discussion

Nowadays, induction of contraction following cervical ripening is widely applied in clinical labor induction. Nevertheless, the labor process cannot be accelerated in cases with insufficient cervix maturity; in contrast, it is likely to increase the risk of fetal intrauterine distress due to the reduction of placental blood perfusion during contractions. In addition, patients are exposed to increased pain caused by contractions [6].

Induction by cervical water balloon placement is a commonly used mechanical approach to promote cervical maturity. Compared with conventional strategies such as prostaglandins, cervical water balloon exhibits a range of advantages, such as causing no uterine overstimulation, higher fetal safety, requiring less clinical supervision, and economic benefits [7]. However, the efficacy of induction depends on the induction method, and individual patient characteristics should be considered as alternative factors affecting the outcome of induction.

Therefore, this study systematically evaluated the effects of multiple risk factors on high-volume Foley catheter-assisted labor induction. These risk factors included age, height, weight, BMI during labor, gravidity, parity, gestational week, uterine height, abdominal circumference, cervical Bishop score, vertex presentation, birth weight, and the indications of induction of labor. Both the multivariate regression equation and logistic model provided convincing results on the predictive accuracy of the induction. The findings are expected to be applied in clinical screening of potential parturients with a low success rate of balloon-induced labor.

The results of univariate regression analysis showed that pregnant women’s height, gravidity, and parity can increase the success rate of labor induction. On the other hand, maternal age, weight, labor-feeding BMI, uterine height, abdominal circumference, and HDP are likely to exhibit a negative effect on labor induction. Considering that cesarean delivery could increase maternal risk [8, 9], labor induction improves the success rate and promotes vaginal delivery. In the present study, pregnant women who eventually underwent cesarean section were defined as a failure of labor induction [10, 11].

The relationship between various risk factors and outcomes of labor induction was analyzed. The results showed that maternal age, weight, BMI, uterine height, and abdominal circumference of vaginal delivery women were generally lower than those who underwent cesarean section. In contrast, the average height of vaginal delivery women was significantly higher. Pregnant women with high cervical Bishop scores or higher gravidity and parity showed remarkable superiority in labor induction. For pregnant women with HDP, the proportion of cesarean sections was significantly higher when utilizing a water balloon for induced labor. These results were consistent with the influencing factors identified in the univariate logistic regression analysis.

The cervical Bishop score before labor induction showed no statistically significant difference between groups and was not included in the regression equation. This may be due to the fact that the cases included in this study were all cases of immature cervix, with Bishop scores of around 4. Indeed, a systematic retrospective study revealed that the cervical Bishop score should not be used to predict the effect of labor induction [12]. Although subjective errors cannot be eliminated, the Bishop score represents the main tool to evaluate the cervical condition before labor induction and assess the changes in cervical maturity after labor induction. In practice, the same medical staff should assess cervical maturity before and after labor induction to reduce the aforementioned error. Moreover, vaginal ultrasound can be used to evaluate the cervical conditions to improve the predictive value [13, 14].

According to the OR values, the parity of pregnant women had the greatest impact on labor induction, while HDP had the smallest. Pregnant women with HDP may be exposed to a higher risk of maternal and fetal complications, such as placental abruption, fetal distress, postpartum hemorrhage, and even intracranial hemorrhage. Pain stimulation during delivery may further increase their blood pressure. Hence, a cesarean section was performed to terminate the pregnancy.

In this study, age, height, BMI, and parity were entered into the logistic regression equation, which was in good accordance with previous results [15–18]. Rossi et al. reported a cesarean section rate of 19.2% among women receiving induced labor [16], which is slightly higher than our data (16.45%). The variables in the equation included previous cesarean section and maternal race. However, these two variables were not included in the current study due to technical and regional limitations.

Among the four variables mentioned above, parity exerted the most significant impact on labor induction. Undoubtedly, the soft delivery canal of multiparous has better compliance and cervical dilation is more easily achieved compared to primiparous women. Height is the second major factor affecting labor induction, which reflects the size of the pelvis. In short pregnant women, the pelvis may be relatively smaller, resulting in an increased head-to-pelvis proportion and a higher failure rate of labor induction.

According to previous reports [17], the increase in maternal age results in decreased endogenous estrogen level, estrogen receptor level, and estrogen sensitivity. Furthermore, the lower compliance of uterine smooth muscles decreases the success rate of labor induction. Pregnant women with high BMI could have fat accumulation in the pelvis and vagina. In addition, due to the relatively small amount of exercise, the pelvic floor muscles are relatively weak, which affects the decline of vertex presentation and increases the rate of cesarean section. Another study showed that [18] among Grade III obese women who received labor induction, the cesarean section rate was nearly 50%. It should be noted that BMI is a risk factor that can be “changed” through long-term management during pregnancy.

## Conclusions

In summary, a nomogram based on logistic multifactor analysis was generated to provide a pre-clinical evaluation tool prior to induction. This tool might be helpful for prenatal communication between doctors and patients and can also be used in clinical discussions. However, this study was single-center research and the nomogram has not been verified externally. Further studies are needed to examine the predictive accuracy in different regions and centers.

Notably, the factors that affect the outcome of induced labor are multifaceted and uncertain. Even though we can reduce the impact of subjective and one-sided factors to some extent by using mathematical models, no predictive value can be provided to recommend cesarean section without vaginal trial labor. Therefore, the decision to perform a cesarean section should be made based on the individual circumstances of each patient and in consultation with the patient and their healthcare provider.

## Data Availability

The datasets used and analysed during the current study available from the corresponding author on reasonable request.
